# MPO-DNA Complexes and cf-DNA in Patients with Sepsis and Their Clinical Value

**DOI:** 10.3390/biomedicines12102190

**Published:** 2024-09-26

**Authors:** Danmei Zhang, Jin Guo, Chunxia Shi, Yukun Wang, Yanqiong Zhang, Xiaoya Zhang, Zuojiong Gong

**Affiliations:** Department of Infectious Diseases, Renmin Hospital of Wuhan University, Wuhan 430060, China; zdm19980503@163.com (D.Z.); 13872214529@163.com (J.G.); 18437950313@163.com (Y.W.); zhangyanqiong217@163.com (Y.Z.); zxy6962023@163.com (X.Z.)

**Keywords:** neutrophil extracellular traps, sepsis, SOFA scores, organ damage

## Abstract

**Background/Objectives:** Neutrophils, as the first line of defense in the immune response, produce neutrophil extracellular traps (NETs) upon activation, which are significant in the pathogenesis and organ damage in sepsis. This study aims to explore the clinical value of myeloperoxidase-DNA (MPO-DNA) and cell-free DNA (cf-DNA) in sepsis patients. **Methods:** Clinical data were collected from 106 sepsis patients, 25 non-sepsis patients, and 51 healthy controls. Sequential Organ Failure Assessment (SOFA) scores were calculated, and levels of MPO-DNA) complexes and cf-DNA were measured using specific kits. Correlation analyses assessed relationships between indicators, while logistic regression identified independent risk factors. Receiver operating characteristic (ROC) curves calculated the area under the curve (AUC) to evaluate the diagnostic value of the biomarkers. **Results:** Sepsis patients exhibited significantly elevated levels of MPO-DNA and cf-DNA compared to non-sepsis patients and healthy controls. In sepsis patients, MPO-DNA and cf-DNA levels correlated with inflammation, coagulation, and organ damage indicators, as well as procalcitonin (PCT) levels and SOFA scores. Both C-reactive protein (CRP) and cf-DNA were identified as independent risk factors for sepsis, demonstrating moderate diagnostic value. ROC analysis showed that the combination of MPO-DNA and CRP (AUC: 0.837) enhances the AUC value of CRP (0.777). **Conclusions:** In summary, elevated serum levels of MPO-DNA and cf-DNA in sepsis patients correlate with SOFA scores and PCT levels, providing reference value for sepsis diagnosis in clinical settings.

## 1. Introduction

Sepsis is a complex clinical condition caused by dysregulated immune responses to infection, often leading to acute organ dysfunction [1]. It is characterized by high morbidity and mortality rates [2]. Despite significant advancements in understanding the host immune response and ongoing research into immunotherapeutic trials for sepsis, these insights have yet to translate into new therapies [3]. Currently, patient stratification based on precisely defined immune phenotypes using biomarkers and molecular mechanisms offers a new approach to the application of immunotherapy in treating sepsis [4]. Therefore, identifying new immune markers and studying patient stratification may provide new directions for the diagnosis and therapeutic management of sepsis.

From the current definition of sepsis, a “dysregulated host response to infection” is a key driving force behind life-threatening organ failure [1]. The host immune response against invading pathogens at the onset of infection is essential for eradicating these pathogens. However, in sepsis, the dysregulation of this response can lead to an excessive inflammatory reaction, resulting in potential organ damage and immune suppression, which is associated with an increased risk of secondary infection and poor prognosis [5,6]. Coagulopathy and severe organ failure [7] are significant outcomes of a dysregulated immune response. Up to one-third of patients with sepsis develop disseminated intravascular coagulation (DIC), characterized by low platelet counts, high levels of fibrin degradation products or soluble fibrin, prolonged clotting times, and reduced endogenous anticoagulant factors [8]. Severe DIC can lead to both thrombosis and excessive bleeding, increasing the risk of death.

Neutrophils are one of the early immune cells responding to pathogen invasion [9]. Neutrophil extracellular traps (NETs) represent a novel mechanism by which neutrophils exert their immune functions and are a crucial component of innate immunity [10]. NETs are released by activated neutrophils and consist of a DNA scaffold, histones, and granular proteases, forming a web of DNA and antimicrobial proteins aimed at trapping and killing pathogens [11]. However, increasing evidence suggests that excessive formation and secretion of NETs contribute to the progression of sepsis, leading to inflammation and tissue damage [12]. Existing studies indicate that NETs can degrade the anticoagulant system and upregulate tissue factors, inducing endothelial cells to exhibit pro-inflammatory and pro-coagulant phenotypes, thereby acting as a bridge between inflammation and coagulation [13]. Furthermore, a study on lung injury demonstrated that patients with infectious acute respiratory distress syndrome (ARDS) and those with cardiac-induced respiratory dysfunction exhibited elevated NET levels. Moreover, there is a correlation between the severity of ARDS, mortality rates, and serum NET levels [14].

Research indicates that soluble NET residues exist in the form of cell-free DNA (cf-DNA) in supernatants in vitro and in serum or tissue fluids in vivo [15]. Free DNA (cell free DNA, cf DNA) is a small non-cellular double-stranded molecule. Soluble NET residues can exist in the form of cf-DNA in supernatant fluid in vitro and serum or tissue fluid in vivo. Previous studies have shown that sepsis patients with higher levels of cf-DNA have lower survival rates and can be used as a predictor of poor prognosis [16]. Furthermore, infection was associated with higher cf-DNA concentrations in critically ill patients [17]. Although cf-DNA is considered to be a marker molecule for NETs, it is not a molecule specific to NETs and may also be secreted from apoptotic or necrotic cells [18]. Another marker molecule of NETs is the myeloperoxidase–DNA (MPO-DNA) complex, which is mainly produced during NETosis. Unlike citrullinated histones, it does not rely on the activation of the peptidylarginine deiminase 4 (PAD4) enzyme required for histone citrullination and is a commonly used marker molecule for NETs [19]. Previous studies have shown that in patients with septic shock, high MPO-DNA levels are closely related to markers of 28-day mortality and severity of organ dysfunction, suggesting that NET formation is an important mechanism causing septic shock [17].

In this study, MPO-DNA complexes and cf-DNA were selected as marker molecules of NETs to explore the changes in NET levels in sepsis patients. The study aims to preliminarily investigate the relationship between MPO-DNA, cf-DNA and the severity of sepsis, as well as their potential role in disease diagnosis.

## 2. Materials and Methods

### 2.1. Study Population

This retrospective study included a total of 182 subjects from Renmin Hospital of Wuhan University between June 2023 and July 2024, comprising 106 patients with sepsis, 51 healthy controls, and 25 non-sepsis patients. The collected clinical data included age, gender, white blood cell count (WBC), neutrophil percentage (Neu%), red blood cell count (RBC), hemoglobin (Hb), platelet count (PLT), procalcitonin (PCT), C-reactive protein (CRP), serum amyloid A (SAA), prothrombin time (PT), international normalized ratio (INR), prothrombin activity (PTA), creatinine (Cr), estimated glomerular filtration rate (eGFR), urea (Ur), uric acid (UA), PaO_2_/FiO_2_ ratio, alanine aminotransferase (ALT), aspartate aminotransferase (AST), albumin (ALB), total bilirubin (TBil), direct bilirubin (DBil), and other parameters, as well as clinical information related to Sequential Organ Failure Assessment (SOFA) scores. The corresponding SOFA scores were calculated. This study was approved by the Ethics Committee of the Renmin Hospital of Wuhan University and was confirmed to exempt patients from informed consent.

Inclusion criteria for the sepsis group were a SOFA score of ≥2 and definite infection, and exclusion criteria were patients under 18 years of age, pregnant, suffering from tumors, and the presence of non-infection-induced organ dysfunction. The non-sepsis group was patients with inflammatory infections that did not meet the requirements for sepsis, and the exclusion criteria were the same as those for the sepsis group. The control group consisted of subjects who underwent physical examination at the same time, excluding those under 18 years of age, those with organ damage, those who had an infection in the previous month, and those who had received antimicrobials or other non-prophylactic medications.

### 2.2. Sample Preparation

Venous blood was collected from patients on admission, and serum was collected after centrifugation of anticoagulant-free blood and stored in an ultra-low temperature refrigerator at −80 °C for subsequent experiments.

### 2.3. Serum MPO-DNA, Cell-Free DNA (cf-DNA) Assay

Serum MPO-DNA was detected by the Human NETs (MPO-DNA) enzyme immunoassay kit (Shanghai Jianglai Biotechnology, Shanghai, China). Pre-coated anti-MPO monoclonal antibody 96-well plates were prepared and placed in a refrigerator at 4 °C overnight. The plates were subsequently closed with 1% BSA at room temperature. Buffer containing peroxidase-labeled anti-DNA antibody was added to the plates. The color was developed in the dark for 30 min using TMB substrate buffer and the absorbance was measured at 450 nm. The concentration of the serum NETs marker cf-DNA was measured using the Quant-iT™ PicoGreen^®^ dsDNA kit (Invitrogen, Waltham, MA, USA). Following the manufacturer’s instructions, DNA standards were diluted to various concentrations using 1× buffer. Briefly, 100 μL of either sample or standard was added to each well of a plate, followed by the addition of 100 μL of Quant-iT PicoGreen working solution. The mixture was thoroughly mixed and incubated at room temperature in the dark for 5 min. Detection was carried out with excitation at 480 nm and emission at 520 nm. All reagents used for testing were utilized within the time frame specified in the instruction manual, and all relevant experimental procedures were strictly followed. The personnel conducting the measurements were blinded to the grouping and allocation of serum samples.

### 2.4. Statistical Analysis

Statistical analysis was performed using SPSS 27.0, GraphPad Prism 8.0, R software 4.3.3 and MedCalc 20. The Kolmogorov–Smirnov test was used to determine whether continuous variables followed a normal distribution. Normally distributed data were expressed as mean ± standard deviation and analyzed using independent sample T-tests or one-way analysis of variance (ANOVA). Non-normally distributed data were expressed as quartiles and analyzed using non-parametric tests. Qualitative data were analyzed using the chi-square test. The correlation between the two variables was assessed using the Pearson or Spearman correlation coefficient. Univariate and multivariate regression analyses were employed to identify independent risk factors. Spearman correlation coefficients and variance inflation factor (VIF) values were used to assess multicollinearity among the variables included in the regression model, with a correlation coefficient > 0.7 indicating high correlation between variables and a VIF > 10 serving as the criterion for determining multicollinearity. Additionally, the diagnostic performance of each indicator was evaluated using the receiver operating characteristic (ROC) curve and the area under curve (AUC) was compared by using Delong’s method. The Hosmer–Lemeshow test assessed the goodness of fit of the model. Decision curve analysis was performed to determine the corresponding clinical value. *p* < 0.05 was considered statistically significant.

## 3. Results

### 3.1. Characteristics of Study Population

Clinical data for all subjects are presented in Table 1. There were no statistically significant differences in gender and age among the groups (*p* > 0.05). The levels of WBC, RBC, Hb, and SAA in both the sepsis group and the non-sepsis group were significantly higher than those in the healthy control group (*p* < 0.05). Additionally, the levels of Neu%, PCT, CRP, AST, DBiL, Cr, eGFR, and Ur in the sepsis group were significantly higher than in the non-sepsis group, while the levels of PLT and ALB were significantly lower (*p* < 0.05).

**Figure 1:**
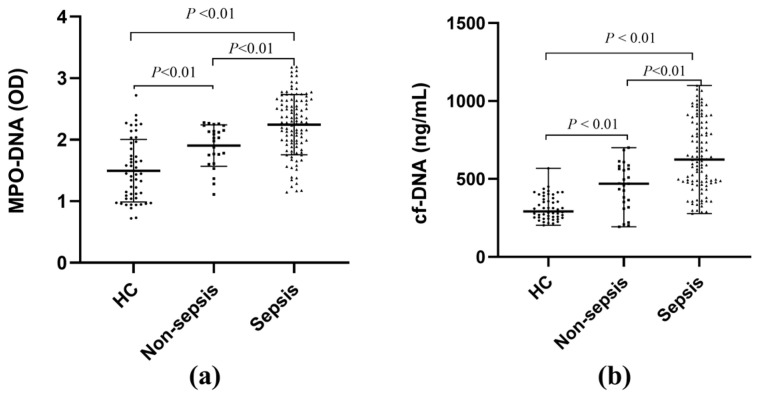
The levels of MPO-DNA and cf-DNA in patients with sepsis and non-sepsis and healthy controls. (**a**) Serum MPO-DNA levels in different groups. (**b**) Serum cf-DNA levels in different groups.

To investigate whether NET formation changes in sepsis patients, we measured the levels of the NET markers MPO-DNA and cf-DNA in the three groups. The results showed that the levels of these two markers were significantly higher in the sepsis group compared to the non-sepsis and healthy control groups, and the levels in the non-sepsis group were significantly higher than those in the healthy control group (Figure 1). These findings suggest that the formation of MPO-DNA and cf-DNA may be involved in the pathogenesis of sepsis.

### 3.2. The Correlations of MPO-DNA and cf-DNA with PCT 

**Figure 2:**
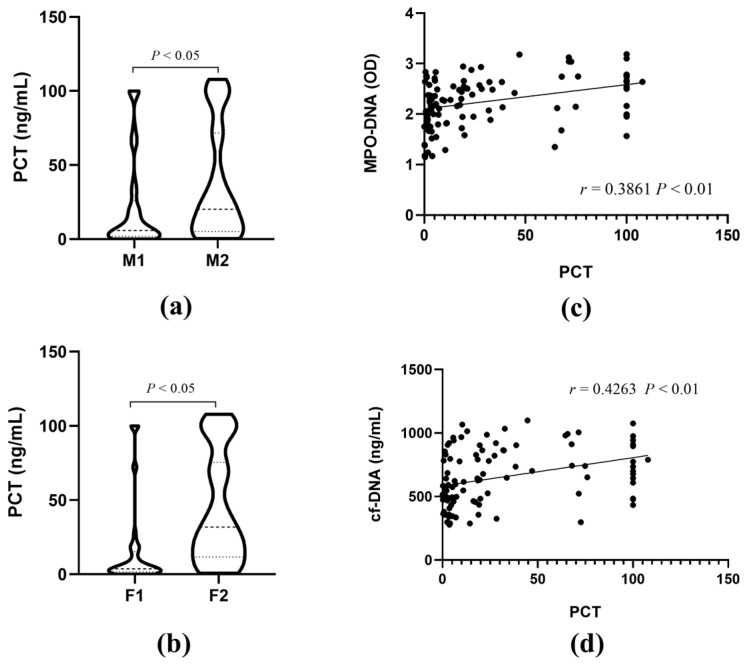
The correlations of MPO-DNA and cf-DNA with PCT. (**a**,**b**) Comparison of PCT levels among patients with different levels of MPO-DNA and cf-DNA. (**c**,**d**) Spearman correlation analysis of PCT with MPO-DNA and cf-DNA.

As we all know, PCT is one of the classic marker molecules of sepsis. To explore the relationship between MPO-DNA levels and PCT, we divided sepsis patients into MPO-DNA low-level group M1 (<2.25) and high-level group M2 (>2.25) based on the average MPO-DNA level of the sepsis patients (Table 1). The PCT levels were then compared between the two groups, and the results showed that the PCT levels in the high-level MPO-DNA group were significantly higher than those in the low-level MPO-DNA group (Figure 2a). Similarly, sepsis patients were divided into cf-DNA low-level group F1 (<625 ng/mL) and high-level group F2 (>625 ng/mL) based on the median cf-DNA value of septic patients (Table 1). The PCT levels were then compared between the two groups, and the results showed that the PCT level in the high-level group (F2) was significantly higher than that in the F1 group (*p* < 0.05) (Figure 2b).

In order to explore the correlation between MPO-DNA and cf-DNA and PCT levels, we used Spearman correlation analysis. The results showed that MPO-DNA and cf-DNA showed obvious positive correlation with PCT, and the correlation coefficients were 0.3861 and 0.4263, respectively (*p* < 0.01) (Figure 2c,d).

### 3.3. The Correlations of MPO-DNA and cf-DNA with Sepsis Severity

**Figure 3:**
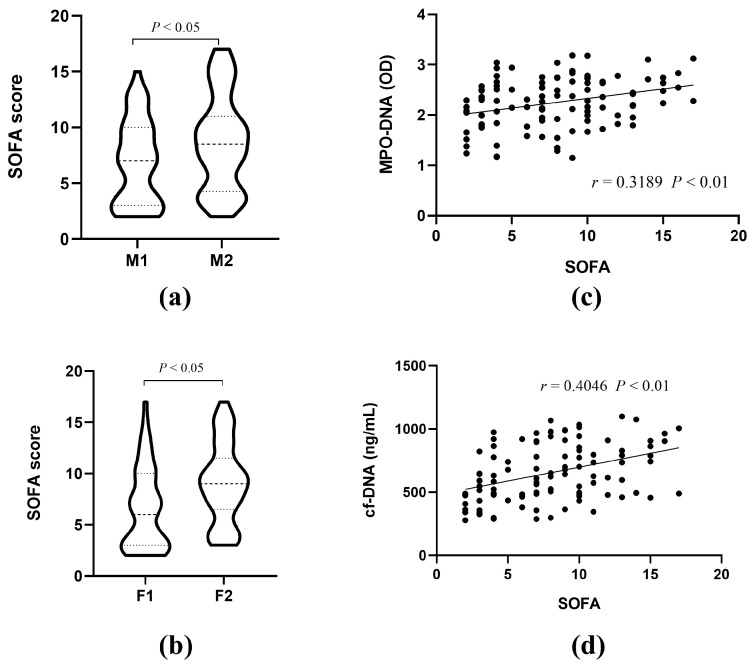
The correlations of MPO-DNA and cf-DNA with sepsis severity. (**a**,**b**) Comparison of SOFA scores among patients with different levels of MPO-DNA and cf-DNA. (**c**,**d**) Spearman correlation analysis of SOFA scores and MPO-DNA and cf-DNA.

The SOFA scoring system is the latest diagnostic standard for sepsis and is related to the severity of critical illness. Higher SOFA scores indicate more severe conditions [20]. To investigate the correlation between NET formation and sepsis severity, sepsis patients were similarly divided into high- and low-level groups based on MPO-DNA and cf-DNA levels, and SOFA scores were compared between the two groups separately. The results showed that SOFA scores were significantly higher in both the high MPO-DNA and high cf-DNA groups compared to the low-level groups, suggesting that NET levels are significantly elevated in severe sepsis patients (Figure 3a,b). Spearman correlation analysis further revealed a significant positive correlation between MPO-DNA, cf-DNA, and SOFA scores (Figure 3c,d).

### 3.4. Correlation Analysis between MPO-DNA and cf-DNA Levels and Clinical Indicators

To explore the correlation between NET formation and clinical indicators, Pearson or Spearman correlation analysis was used to assess the relationship between MPO-DNA and cf-DNA levels and organ injury, coagulation function, and inflammatory indicators in sepsis patients. The results (Table 2) showed that MPO-DNA levels were correlated with liver injury indicators (ALB, DBiL) and myocardial injury indicators (N-terminal pro-brain natriuretic peptide (NT-proBNP), cardiac troponin (cTnI), and Creatine Kinase, MB Form (CK-MB)). Both MPO-DNA and cf-DNA levels were significantly correlated with kidney injury indicators (Cr, eGFR, Ur, and UA), coagulation function indicators (PT, INR, and PTA%), and inflammatory indicators (Neu%, PCT). These results indicate that the abnormal formation of MPO-DNA and cf-DNA is associated with inflammatory pathological processes and multi-organ injury to some extent.

### 3.5. Diagnostic Value of MPO-DNA and cf-DNA in Sepsis

To explore the diagnostic roles of MPO-DNA and cf-DNA in sepsis versus non-sepsis, we conducted a logistic regression analysis on these two patient groups. The results indicated that Neu (%), PCT, CRP, SAA, MPO-DNA, and cf-DNA were significant influencing factors for sepsis (*p* < 0.05). Further multivariate logistic regression analysis revealed that CRP and cf-DNA were independent risk factors for sepsis (*p* < 0.05) (Table 3), demonstrating moderate diagnostic value. Moreover, correlation and collinearity analyses showed that the correlation coefficients among CRP, MPO-DNA, and cf-DNA were <0.5, with tolerance > 0.1 and VIF < 2, indicating the absence of collinearity among these three independent variables (Figure S1, Table S1).

**Figure 4:**
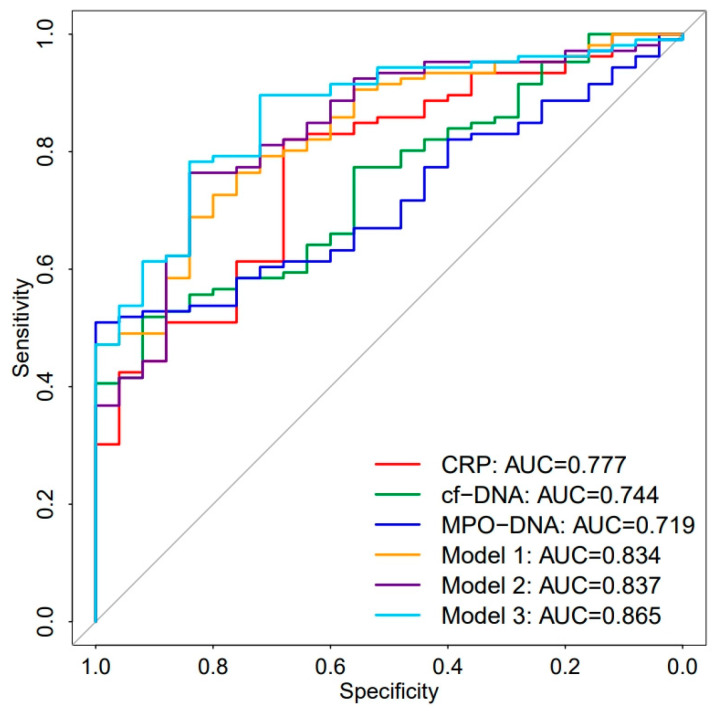
ROC curves for different parameters and models in the diagnosis of sepsis (Model 1: CRP + cf-DNA, Model 2: CRP + MPO-DNA, Model 3: Model I + MPO-DNA).

**Figure 5:**
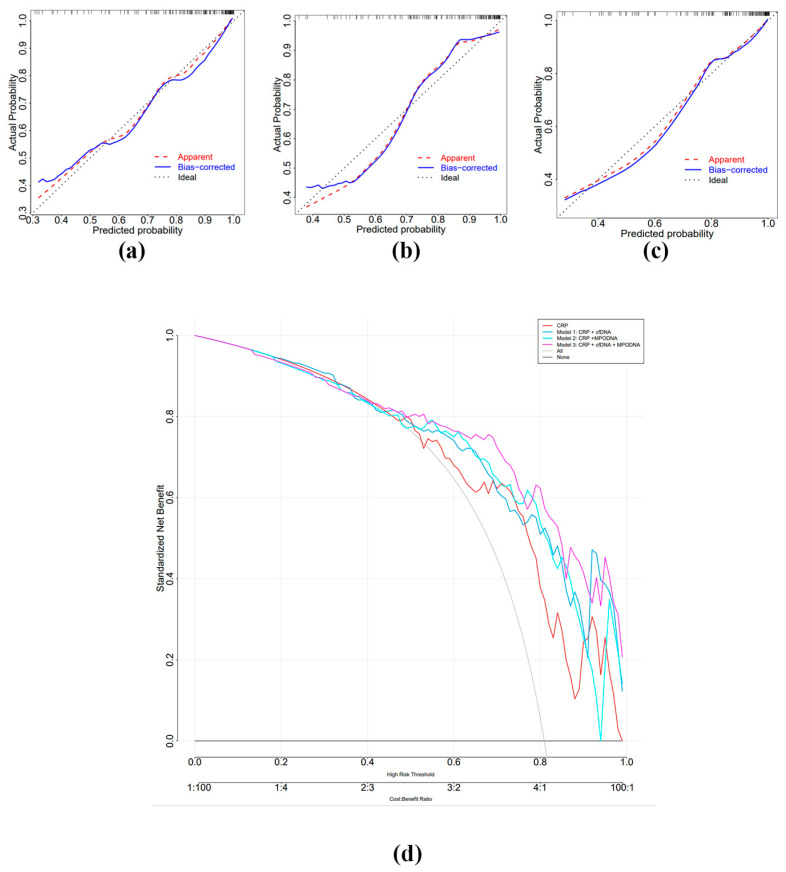
Calibration curves and decision curves for evaluating the performance of Models 1–3. (**a**) Calibration curve for Model 1, (**b**) calibration curve for Model 2, (**c**) calibration curve for Model 3, (**d**) decision curve analysis for CRP and Models 1–3.

Although MPO-DNA was not identified as an independent risk factor for sepsis, we still generated an ROC curve for it due to its suggestive role in the prognosis of sepsis [17]. The results showed that the AUC values for CRP, cf-DNA, and MPO-DNA were 0.777 (95% CI: 0.680–0.873), 0.744 (95% CI: 0.650–0.838), and 0.719 (95% CI: 0.628–0.809), respectively (Figure 4, Table 4). While CRP had the highest AUC value, there were no statistically significant differences among them (Table S2). To further investigate whether the inclusion of MPO-DNA and cf-DNA could improve the AUC value of CRP, we constructed Model 1 (CRP + cf-DNA) based on the results of the multivariate logistic regression analysis. Additionally, we created two models by incorporating MPO-DNA into CRP and Model 1 (Model 2: CRP + MPO-DNA, Model 3: Model 1 + MPO-DNA). The AUC values for these three models were 0.834 (95% CI: 0.759–0.893), 0.837 (95% CI: 0.762–0.895), and 0.865 (95% CI: 0.795–0.919), respectively (Figure 4, Table 4). Subsequently, we performed the Hosmer–Lemeshow goodness-of-fit test and constructed decision curves for the three models. The results demonstrated a good agreement between the predicted probabilities and the observed probabilities for all three models (Hosmer–Lemeshow goodness-of-fit test: Model 1: *p* = 0.9865, Model 2: *p* = 0.1937, Model 3: *p* = 0.9747) (Figure 5a–c). The decision curve analysis indicated that all three models provided net benefits across most thresholds, consistently outperforming CRP alone (Figure 5d). All three models exhibited strong performance. Through DeLong’s test analysis, we found that the AUC value of Model 2 showed a significant improvement over CRP alone, with statistical significance (*p* < 0.05), indicating that the addition of MPO-DNA could enhance the diagnostic value of CRP and provide diagnostic insight. Furthermore, the inclusion of MPO-DNA in Model 1 also significantly improved the diagnostic value of CRP to a certain extent (*p* < 0.05) (Table S2).

## 4. Discussion

Sepsis is a common and fatal complex disease in clinical practice. Despite improvements in medical conditions, the incidence and mortality rates of sepsis have slightly declined but it remains a major cause of global health damage [21]. Approximately 30–50% of inpatient deaths are associated with sepsis [22]. A cytokine storm triggered by dysregulated immune responses is a crucial factor in sepsis-induced multi-organ damage [23]. Multiple studies have demonstrated that abnormally formed neutrophil extracellular traps (NETs) can interact with endothelial cells, further exacerbating immune responses; on the other hand, they directly damage organs, further promoting sepsis-induced organ injury [24]. Therefore, exploring the role of NETs in the pathogenesis of sepsis provides new directions for precise immunotherapy and improving sepsis outcomes.

During sepsis, the interaction between neutrophils and endothelial cells is enhanced, promoting neutrophil infiltration into tissues, which in turn increases NET formation [25]. Our study results indicate that the elevated levels of cf-DNA in sepsis patients align with previous research findings. During sepsis, cf-DNA can originate from multiple sources, including the occurrence of NETosis and the release of NETs, apoptosis or necrosis of cells, and the destruction of pathogens [26]. Therefore, circulating cf-DNA in sepsis patients may not solely originate from the formation and release of NETs. In this regard, circulating MPO-DNA complexes are more specific to NETs than cf-DNA, which is related to the formation mechanism of MPO-DNA. Although previous study showed that using the ELISA method to detect MPO-DNA complexes in the patients’ plasma was prone to errors [27], advances in technology have led to an increasing number of studies utilizing ELISAs to measure circulating MPO-DNA levels in patients [28,29,30]. Similarly, our study found that MPO-DNA levels were significantly higher in sepsis patients compared to non-sepsis patients and healthy controls. Additionally, MPO-DNA and cf-DNA levels were significantly higher in non-sepsis patients than in healthy controls, indicating that abnormal NET formation may also occur in general inflammatory diseases. Previous studies have shown that elevated MPO-DNA levels in the plasma are associated with transfusion-related acute lung injury, ANCA-associated small vessel vasculitis [31], acute liver failure [32], severe coronary atherosclerosis [33], and thrombotic microangiopathies [34] related to their pathogenic mechanisms. Moreover, our correlation analysis found that the levels of MPO-DNA and cf-DNA exhibited low to moderate correlations with clinical indicators. Specifically, MPO-DNA correlated to varying degrees with the percentage of neutrophils, kidney injury, myocardial injury, and coagulation function indicators. The elevated levels of these markers were positively correlated with the severity of organ damage. Previous research has indicated that abnormal NET formation can activate platelets and promote thrombosis. Another study suggested that although MPO-DNA levels were not correlated with DIC scores, they were elevated in patients with DIC-related septic shock [17]. This indicates that the abnormal elevation of circulating MPO-DNA levels may be involved in the progression of coagulation abnormalities in sepsis patients, promoting abnormal thrombosis formation. Consistent with this, a clinical study also demonstrated that cf-DNA levels are directly related to multiple organ dysfunction scores, sepsis-related organ function assessment, white blood cell count, and MPO levels [35]. Additionally, a study involving 55 critically ill patients showed that MPO-DNA complex levels correlate with the severity of organ dysfunction and 28-day mortality [17].

PCT is one of the most commonly used indicators for sepsis diagnosis, medication guidance, and prognosis [36]. Under normal conditions, PCT levels in the human body are extremely low, but they rapidly increase during infection [37]. The SOFA scoring system is the latest diagnostic standard for sepsis, as specified in the new sepsis definitions, and is related to the severity of the condition in critically ill patients. A higher SOFA score indicates a more severe condition. To explore the relationship between NET levels, the severity of sepsis, and PCT levels in sepsis patients, we divided the sepsis patients into high- and low-concentration groups based on the average levels of the NET marker MPO-DNA and the median cf-DNA levels. The results showed that in both the high-level MPO-DNA group and the high-level cf-DNA group, PCT levels and SOFA scores were higher than those in the low-level groups. Similarly, Spearman correlation analysis showed that MPO-DNA and cf-DNA levels were positively correlated with PCT and SOFA scores to varying degrees, suggesting that abnormal NET formation plays an important role in the progression of sepsis. Some studies have also indicated that MPO-DNA and cf-DNA levels can predict the prognosis and organ dysfunction in patients with septic shock to some extent. Furthermore, abnormal NET formation is also associated with poor prognosis in patients with acute liver failure [32].

Research on NETs in sepsis is becoming increasingly in-depth [38]. Studies using the CLP-induced sepsis model have found that intervening in NET formation during the late rather than early stages of sepsis can alleviate inflammation and increase survival rates [39]. Novel intervention strategies targeting NETs are also under continuous investigation [14,40,41]. However, research on the diagnostic value of NETs in sepsis remains insufficient. The results of this study indicate that CRP and cf-DNA are independent risk factors for sepsis in both the sepsis and non-sepsis groups, demonstrating moderate diagnostic value. Although MPO-DNA was not identified as an independent risk factor, previous studies have suggested that MPO-DNA has certain prognostic value in sepsis and organ failure [17]. Furthermore, MPO-DNA has shown diagnostic value in anti-neutrophil cytoplasmic antibody-associated vasculitis. Therefore, we generated an ROC curve for MPO-DNA and found that the AUC values among these three indicators did not show statistically significant differences, all exhibiting moderate diagnostic significance. To further investigate the diagnostic significance of MPO-DNA and cf-DNA, we combined cf-DNA, selected through multivariate logistic regression, with CRP to create Model 1. Subsequently, we incorporated MPO-DNA into CRP and Model 1 to construct Models 2 and 3, respectively, and generated ROC curves. The results indicated that the inclusion of MPO-DNA, rather than cf-DNA, somewhat improved the AUC value of CRP, suggesting that MPO-DNA has a certain diagnostic indicatory role.

Nonetheless, this study has several limitations. Firstly, it is restricted to a small sample size, and further validation with larger sample sizes is needed. Additionally, future studies should further stratify sepsis and septic shock groups to verify the relationship between NETs and sepsis severity and further track prognosis to explore the relationship between NET levels and sepsis outcomes.

In summary, our study indicates that the levels of MPO-DNA and cf-DNA are significantly elevated in sepsis patients and are associated with multi-organ damage and inflammation. The levels of MPO-DNA and cf-DNA are associated with the SOFA scores and PCT levels in sepsis patients. Both MPO-DNA and cf-DNA demonstrate certain diagnostic value. When MPO-DNA is combined with CRP, it can enhance the AUC value of CRP to some extent, indicating a significant diagnostic indicative role.

## Figures and Tables

**Table 1:** Patient characteristics.

Variable	Sepsis Group (n = 106)	Non-Sepsis Group (n = 25)	Healthy Controls (n = 51)
Gender (male/female)	49/57	12/13	24/27
Age	64.45 ± 12.30	58.28 ± 15.76	64.63 ± 15.26
WBC (10^9^/L)	13.800 (8.8,18.8) *	9.490 (6.0,14.8) *	6.000 (4.8,7.2)
Neu (%)	89.800 (83.7,93.2) *^,#^	82.800 (70.4,90.0) *	53.900 (46.9,60.2)
RBC (10^12^/L)	3.495 (3.0,4.0) *	3.560 (3.3,4.2) *	4.640 (4.3,4.9)
Hb (g/L)	106.500 (84.8,119.0) *	105.000 (96.0,120.0) *	143.000 (132.0,152.0)
PLT (10^9^/L)	99.500 (57.3,164.0) *,#	247.000 (195.5,272.0)	225.000 (177.0,252.0)
PCT (ng/mL)	13.657 (3.0,45.2) *^,#^	0.970 (0.3,1.9) *	0.035 (0.0,0.1)
CRP (mg/L)	126.030 (75.0,166.1) *^,#^	44.780 (15.7,108.0) *	2.870 (1.8,4.8)
SAA (mg/L)	300.000 (162.0,300.0) *	175.970 (81.9,294.2) *	4.440 (2.0,7.8)
ALT (U/L)	25.500 (12.8,68.3)	24.000 (9.0,29.0)	19.000 (14.0,29.0)
AST (U/L)	41.000 (18.0,88.0) *^,#^	24.000 (13.0,33.0)	21.000 (19.0,27.0)
ALB (g/L)	30.235 (27.8,34.4) *^,#^	33.700 (31.2,37.5) *	45.900 (44.5,47.0)
TBiL (μmol/L)	16.500 (10.3,25.9)	9.000 (7.0,12.4) *	13.300 (11.4,16.8)
DBiL (μmol/L)	7.965 (4.6,15.1) *^,#^	4.700 (2.9,7.6)	4.100 (3.3,5.5)
Cr (μmol/L)	141.500 (74.5,266.3) *^,#^	74.000 (52.0,90.0)	69.000 (55.0,80.0)
eGFR (mL/min)	38.970 (17.0,69.4) *^,#^	87.490 (62.9,103.1)	88.880 (78.5,102.0)
Ur (mmol/L)	13.240 (8.0,20.0) *^,#^	4.635 (3.7,8.6)	5.560 (4.7,6.8)
UA (μmol/L)	378.000 (263.3,467.3) ^#^	246.000 (182.0,356.5) *	326.000 (292.0,388.0)
cf-DNA (ng/mL)	624.990 (478.7,857.9) *^,#^	469.966 (335.0,573.0) *	292.328 (253.5,376.3)
MPO-DNA (OD)	2.25 ± 0.49 *^,#^	1.91 ± 0.34 *	1.50 ± 0.51

Notes: Significant differences are presented as * *p* < 0.05, compared with the control group; ^#^ *p* < 0.05, compared with non-sepsis group.

**Table 2:** The correlation between MPO-DNA, cf-DNA, and organ damage markers and inflammatory indexes.

Variables	MPO-DNA	cf-DNA
WBC (10^9^/L)	0.006	0.025
N (%)	0.243 *	0.217 *
PLT (10^9^/L)	−0.211 *	−0.314 **
PCT (ng/mL)	0.386 **	0.426 **
CRP (mg/L)	0.216 *	0.173
SAA (mg/L)	0.168	0.113
ALT (U/L)	−0.045	0.124
AST (U/L)	0.05	0.165
ALB (g/L)	−0.210 *	−0.158
TBiL (μmol/L)	0.1	0.133
DBiL (μmol/L)	0.200 *	0.109
Cr (μmol/L)	0.235 *	0.299 **
eGFR (mL/min)	−0.258 **	−0.295 **
Ur (mmol/L)	0.209 *	0.375 **
UA (μmol/L)	0.235 *	0.222 *
PT (s)	0.245 *	0.192 *
INR	0.264 **	0.195 *
PTA (%)	−0.277 **	−0.192 *
NT-proBNP (pg/mL)	0.284 **	0.076
cTnI (ng/L)	0.211 *	0.029
CK-MB (ng/mL)	0.240 *	0.128

Notes: The Pearson correlation method was used to analyze the correlation between MPO-DNA and PTA (%); Spearman correlation analysis was used to calculate the correlation coefficients between MPO-DNA, cf-DNA, and other indicators. Abbreviations: WBC, White blood cell; N (%), Neutrophil percentage; PLT, Platelet; PCT, Procalcitonin; CRP, C-reactive protein; SAA, Serum amyloid A protein; ALT, Alanine aminotransferase; AST, Aspartate aminotransferase; ALB, Albumin; TBiL, Total bilirubin; DBiL, Direct bilirubin; Cr, creatinine; eGFR, Estimated glomerular filtration rate; Ur, Urea; UA, Uric acid; PT, Prothrombin time; INR, International normalized ratio; PTA, Prothrombin time activity; NT-proBNP, N-terminal pro-brain natriuretic peptide; cTnI, Cardiac troponin; CK-MB, Creatine Kinase, MB Form. * *p* < 0.05 ** *p* < 0.01.

**Table 3:** Univariate and multivariate regression analysis of variables for sepsis.

Variables	Univariate Analysis		Multivariate Analysis	
	OR (95% CI)	*p* Value	OR (95% CI)	*p* Value
WBC	0.983–1.110	0.161	/	/
Neu (%)	1.010–1.078	0.011	0.978–1.059	0.390
RBC	0.482–1.348	0.411	/	/
PCT	1.031–1.228	0.008	0.961–1.145	0.288
CRP	1.010–1.029	<0.01	1.005–1.030	0.007
SAA	1.001–1.009	0.029	0.991–1.003	0.337
MPO-DNA	1.739–12.880	0.002	0.912–14.366	0.067
cf-DNA	1.002–1.008	<0.01	1.000–1.007	0.046

**Table 4:** ROC curve analysis for different parameters and models in the diagnosis of sepsis.

Variables	AUC	Sensitivity%	Specificity%	95%CI	Youden Index	Cut-Off
MPO-DNA	0.719	52.8	92	0.628–0.809	0.448	2.261
cf-DNA	0.744	55.7	84	0.650–0.838	0.397	587.679
CRP	0.777	82.1	68	0.680–0.873	0.501	59.515
Model 1	0.834	76.4	76	0.755–0.913	0.524	/
Model 2	0.837	76.4	84	0.753–0.903	0.604	/
Model 3	0.865	78.3	84	0.794–0.936	0.623	/

## Data Availability

Data is contained within the article or Supplementary Materials.

## Supplementary Materials

The following supporting information can be downloaded at: https://www.mdpi.com/article/10.3390/biomedicines12102190/s1, Figure S1: Correlation of variables in Cox regression analysis.; Table S1: The tolerance and VIF for the logistic regression analysis.; Table S2: Pairwise comparisons of different ROC curves between different parameters and Models.
