# Supplementary material for: MPO-DNA Complexes and cf-DNA in Patients with Sepsis and Their Clinical Value
Source: Biomedicines. 2024 Sep 26;12(10):2190. doi: 10.3390/biomedicines12102190 (PMC11505433; doi:10.3390/biomedicines12102190)
Supplement: Supplementary file 1 [file biomedicines-12-02190-s001.zip › biomedicines-3209714-supplementary.pdf]

SUPPLEMENTAL MATERIALS

Supplementary Figure S1: Correlation of variables in Cox regression analysis.
Supplementary Table S1: The tolerance and VIF for the logistic regression analysis.
Supplementary Table S2: Pairwise comparisons of different ROC curves between different parameters and Models.

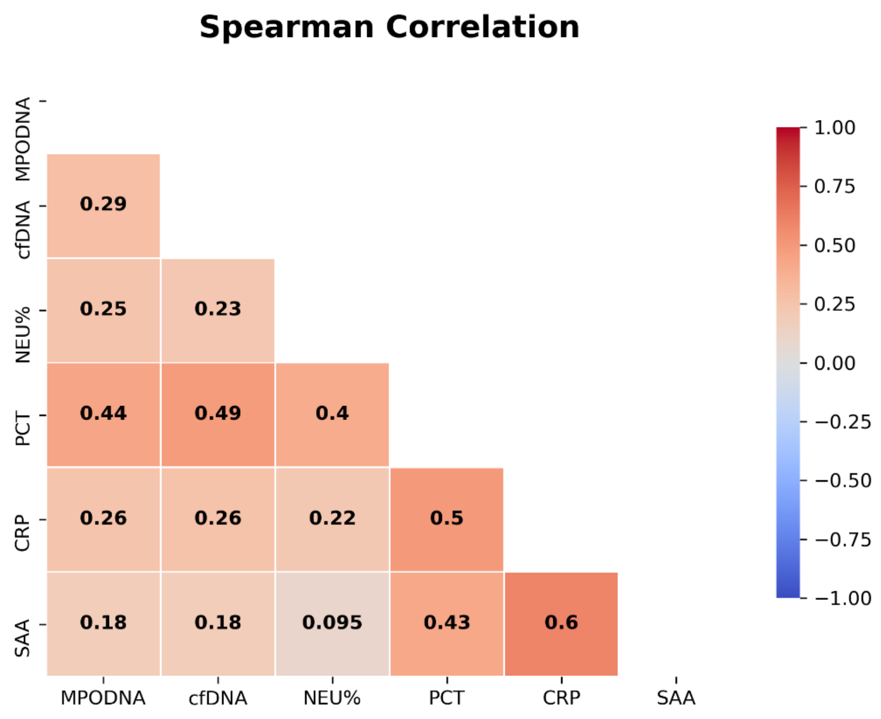

Figure S1. Correlation of variables in Cox regression analysis.

Table S1. The tolerance and VIF for the logistic regression analysis.

Characteristics	Tolerance	VIF
MPO-DNA	0.827	1.210
cf-DNA	0.800	1.249
N (%)	0.881	1.135
PCT	0.608	1.644
CRP	0.571	1.752
SAA	0.646	1.548

Table S2. Pairwise comparisons of different ROC curves between different parameters and Models.

Pairwise comparison	DBA	SE	95 %CI	z statistic	P value
CRP~ cf-DNA	0.0328	0.0669	-0.0982 - 0.164	0.491	NS
CRP~ MPO-DNA	0.0581	0.0687	-0.0765 - 0.193	0.846	NS
cf-DNA ~ MPO-DNA	0.0253	0.0661	-0.104 - 0.155	0.383	NS
Model 1~ CRP	0.0570	0.0293	-0.000416 - 0.114	1.946	NS
Model 1~ cf-DNA	0.0898	0.0427	0.00607 - 0.174	2.102	0.0356
Model 1~MPO-DNA	0.115	0.0613	-0.00509 - 0.235	1.877	NS
Model 2~ CRP	0.0600	0.0600	0.00509 - 0.115	2.142	0.0322
Model 2~ cf-DNA	0.0928	0.0639	-0.0325 - 0.218	1.452	NS
Model 2~ MPO-DNA	0.118	0.0479	0.0241 - 0.212	2.464	0.0138
Model 2~ Model 1	0.00302	0.0338	-0.0631 - 0.0692	0.0894	NS
Models 3~ CRP	0.0887	0.0352	0.0196 - 0.158	2.516	0.0119
Model 3~ cf-DNA	0.122	0.0456	0.0321 - 0.211	2.664	0.0077
Model 3~ MPO-DNA	0.147	0.0461	0.0565 - 0.237	3.186	0.0014
Models 3~ Model 1	0.0317	0.0217	-0.0108 - 0.0742	1.460	NS
Models 3~ Model 2	0.0287	0.0229	-0.0163 - 0.0736	1.251	NS
